# Identifying essential resource parameters for pandemic preparedness and response: an international Delphi study within the EU PANDEM-2 project

**DOI:** 10.1136/bmjopen-2023-079609

**Published:** 2024-12-15

**Authors:** Berend H. H. Beishuizen, Mart L Stein, Joeri S Buis, Alma Tostmann, Caroline Green, James Duggan, Máire A Connolly, Chantal P Rovers, Aura Timen

**Affiliations:** 1 Centre for Infectious Disease Control, National Institute for Public Health and the Environment, Bilthoven, The Netherlands; 2 Department of Primary and Community Care, Radboud University Medical Center, Nijmegen, Gelderland, The Netherlands; 3 Department of Medical Microbiology, Radboud Center for Infectious Diseases, Radboud University Medical Center, Nijmegen, Gelderland, The Netherlands; 4 School of Computer Science and Data Science Institute, University of Galway, Galway, Ireland; 5 School of Health Sciences, College of Medicine, Nursing and Health Sciences, University of Galway, Galway, Ireland; 6 Department of Internal Medicine, Radboud Center for Infectious Diseases, Radboud University Medical Center, Nijmegen, Gelderland, The Netherlands

**Keywords:** Public health, COVID-19, PUBLIC HEALTH

## Abstract

**Abstract:**

**Objective:**

The COVID-19 pandemic highlighted the crucial role of healthcare and public health resource management, where scarcity impairs pandemic response resulting in increased disease transmission, delayed patient care and poorer health outcomes. In the EU PANDEM-2 project, we aimed to identify essential resource parameters for pandemic preparedness and response in the context of an emerging viral respiratory illness.

**Design:**

After performing a systematic literature review, we conducted a Delphi study consisting of a structured questionnaire and consensus round with two separate panels of European public health experts (PHEs) and clinicians, respectively. Resources were categorised as material (n=23), human (n=18) or pharmaceutical (n=12). Data were analysed descriptively for both panels.

**Results:**

Participants were 17 PHEs and 16 clinicians from nine countries. Consensus between the two panels was found on 40 resource parameters (17 material, 14 human, 9 pharmaceutical; 33 accepted and 7 rejected). Notably, clinicians selected three home care resources while PHEs did not, and PHEs selected two pharmaceutical resources which clinicians did not. No consensus was observed on 13 resources. Eleven additional resources were suggested and included (five for PHE and six for clinicians) among which were personal protective equipment for mobile teams, resources for primary care and resources related to mechanical ventilation.

**Conclusions:**

The high level of consensus between the two expert panels indicates common goals in pandemic resource planning. The disagreement on 13 resource parameters reflects the different priorities between PHEs and clinicians in pandemic planning. This study has demonstrated the core components of resource modelling required for pandemic preparedness planning and shows the importance of consulting experts with both public health and clinical backgrounds. Including our proposed resources in pandemic models allows for more enhanced planning and training activities for future pandemics.

**STRENGTHS AND LIMITATIONS OF THIS STUDY**

- This study used the Delphi consensus method to determine the relevance of both public health and healthcare resource parameters for pandemic preparedness, a novel addition to current modelling work which focuses only on clinical care.
- Two separate consensus procedures were conducted, one with public health experts and one with clinicians. The findings highlight both common ground and differences in perspectives.
- Lower retention from questionnaire to consensus procedure may have reduced the diversity of opinion and quality of consensus discussions.
- Due to the ongoing COVID-19 pandemic, the planned consensus discussions were conducted online supported by moderators with experience of the Delphi process and blind online voting tools.

## Introduction

 Infectious disease outbreaks and pandemics have occurred with increasing frequency in recent decades,^1^ and their impact has been more severe in our globally connected world.^2^ The COVID-19 pandemic, since May 2023 no longer considered a pandemic but ‘an ongoing health issue’ by the WHO,^3^ is the most recent example, with an unprecedented impact on society as a whole and health systems in particular. The high infection rates and hospitalisations throughout multiple waves had a significant impact on healthcare resource utilisation. At certain points during the pandemic, especially during the initial waves, imbalances between supply and demand led to acute shortages for patients and healthcare providers, resulting in increased morbidity and mortality. The imbalance between supply and demand led to major delays in the delivery of essential medical countermeasures such as personal protective equipment (PPE) due to worldwide shortages but also at some point shipping containers being held up in ports.^4^ The sudden surge in demand on hospital resources caused by the high transmissibility and pathogenicity of SARS-CoV-2 led to record numbers of patients experiencing delays in healthcare delivery with adverse health outcomes.^5 6^ Public health resources have also been widely deployed and required significant scaling up during the COVID-19 pandemic, largely for testing, contact tracing and vaccination.

Healthcare resources can be loosely defined as the people and items required to keep all parts of the healthcare system (primary care, clinical care, home care and also public health) running. For pandemic preparedness or resource modelling for pandemic preparedness, not only the resource (eg, physicians) but also the associated parameters (eg, number of beds a physician attends, rate of physician absenteeism) are needed to determine the rate at which a resource is used and thus how many are needed to control infectious diseases and maintain care delivery. Financial assets are a vital resource as well, and the cost and shelf-life of resources are important aspects of pandemic resource planning, but neither are part of the scope of this study.

Pandemic preparedness aims to reduce the likelihood of an outbreak and build capacity for effective response and recovery.^7^ Including resource management in this process is of vital importance as effective response and recovery are impossible when sufficient resources are not available in time. Previous research has shown the importance of resource planning and modelling, but published models generally focus on resources for clinical care^8^,^10^ and smaller scale modelling studies generally only include a limited number of resources.^11 12^ Expanding models to include more resources and resource parameters (from here on collectively referred to as ‘resources’), and both clinical care and public health resources, allows the simulation of more realistic scenarios for preparedness exercises. The European Union (EU) PANDEM-2 project^13^ has sought to address this by developing a resource planning system that integrates multiple clinical care and public health resources with an epidemiological model.^14^ This study informed the resource planning system on essential resources and associated parameters that should be taken into account. The PANDEM-2 project further included work on a dashboard and database, simulation exercise scenarios, communication handbooks and a social media analysis tool, all intended to support pandemic preparedness activities.

Bayram *et al*^15^ previously conducted a Delphi consensus procedure on the selection of hospital resources specific to critical care with a panel of hospital professionals. We designed a Delphi consensus procedure for the selection of both public health and clinical care resources and associated parameters and recruited experts from various EU countries who have professional backgrounds in either public health or clinical care. The importance of including participants from varied professional and geographic backgrounds cannot be understated because pandemic preparedness is a multidisciplinary, cross-border activity that requires international cooperation. The aim was to critically evaluate which resource parameters are most important for (1) pandemic preparedness, in general, and (2) resource modelling for pandemic preparedness, in particular.

## Methods

### Study design

This study uses a Delphi consensus procedure^16 17^ consisting of a systematic literature review, a questionnaire and two separate international expert panels (the first conducted in January and February 2022, and the second in May 2022). The Delphi procedure is a well-established methodology using an iterative setup to explore consensus between subject matter experts.^18^ The ACCORD reporting guidelines were used for this study (online supplemental material 1).^19^ A protocol for this study was not prospectively registered. We conducted the Delphi study in the context of an emerging viral respiratory illness, drawing on experience from the COVID-19 pandemic. The literature review was designed to search for public health and healthcare resources included in publications relating to the 2009 H1N1 influenza pandemic and the COVID-19 pandemic. The questionnaire was used to rank the appropriateness of resource parameters for pandemic preparedness planning and modelling and determine which resources participants had already reached a consensus on or where further discussion was required. It also gave participants the opportunity to comment on proposed resource parameters or suggest new resources. Following this, two online consensus rounds were held to discuss those items for which no consensus was reached in the questionnaire, and to discuss additional resources. This study was conducted within the EU PANDEM-2 H2020 project, and panel members were recruited using the network of PANDEM-2 partners.

### Recruitment

Panel members were recruited via purposive snowball sampling: targeting specific individuals for recruitment and asking them to forward the invitation to other experts in their network. We invited partners in the PANDEM-2 project with experience in pandemic preparedness and response to participate in this study, and also asked all partners in the PANDEM-2 project to forward the invitation to stakeholders within their country who were involved in pandemic preparedness and response previously, or during the COVID-19 pandemic.

While initial recruitment was targeted at public health and clinical care professionals, it yielded only public health experts (PHEs), possibly due to COVID-19-related workload during the fall and winter of 2021. We therefore decided to perform one Delphi consensus round with PHEs and a separate second Delphi consensus round specifically with clinicians to ensure both perspectives were represented in this study. To recruit clinicians, we used the PANDEM-2 network, specifically targeting clinicians working in hospitals, primary care or home care who were involved in pandemic planning and response.

### Questionnaire development

A questionnaire (online supplemental material 2) with a core set of 53 resource parameters was developed based on a systematic literature review, which will be published separately, and expert opinion. The resources were categorised into three groups: (1) material resources (n=23), (2) human resources (n=18) and (3) pharmaceutical resources (n=12). Respondents were asked to score each resource using a nine-point Likert scale, ranging from 1 ‘Not at all relevant’ to 9 ‘Extremely relevant’. All resources had to be appraised for relevance to (1) pandemic preparedness and (2) resource modelling for pandemic preparedness. The option to answer ‘I don’t know’ was also provided for each resource. Participants could provide comments in an open text box after each section, and again at the end of the questionnaire. They were also provided an opportunity to suggest additional resources or parameters that were not included in the questionnaire. The questionnaire was piloted twice with a hospital resource management specialist to ensure clarity of the questions.

### Questionnaire validity

We considered construct validity, content validity and face validity when developing the questionnaire. Construct validity was not considered to be an issue in this questionnaire as the items included do not represent or reflect concepts, but instead are real resources. Mouse-over explanations were added to the questionnaire to support construct validity. We piloted the questionnaire for content and face validity, first internally with PHEs and clinicians of diverse backgrounds, and second externally in two rounds with a hospital capacity manager.

### Data analysis

**Figure 1:**
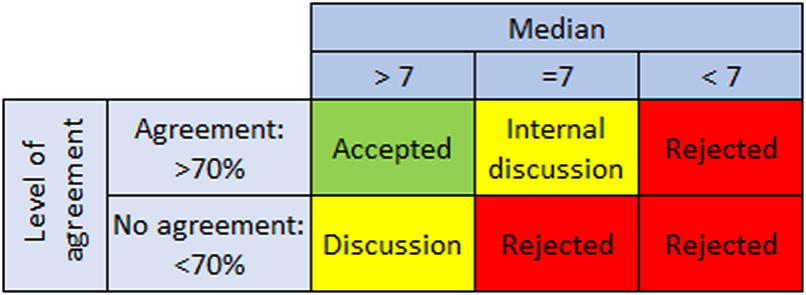
Table with analysis approach of the Delphi questionnaire results. ‘Median score’ indicates the median score on a nine-point Likert scale for a specific resource. ‘Level of agreement’ indicates whether sufficient participants (at least 70%) scored the item in the highest tertile range (7–9) to accept the resource.

The questionnaire results were analysed descriptively to calculate median relevance scores and agreement per resource. A resource was ‘accepted’ directly when the median score was >7 and more than 70% of scores fell in the highest tertile (score: 7, 8 or 9), indicating sufficient agreement. Resources with a median score above 7 but with less than 70% of scores in the highest tertile were designated for further discussion in the consensus procedure, and resources with a median score of 7 and >70% of scores in the highest tertile were discussed within the research team, with possible outcomes being inclusion, discussion in the consensus procedure or rejection. Resources with a median score below 7 were rejected without further discussion.^16^ A schematic overview is given in figure 1.

### Consensus procedures

All respondents to the questionnaire were invited to attend the consensus procedure to discuss resources for which no consensus was reached in the questionnaire. Prior to the consensus round, each participant received a personal feedback report detailing the analysis of the scores, their personal scores for each resource and an overview of the group scores. Within the separate panels, additional resources suggested in open-text fields were added to the report. The consensus meetings were held online using Microsoft Teams due to COVID-19 restrictions and the international nature of the panels. The consensus procedures were chaired by two experts on infectious disease management, one with a background in public health and primary care and the other with a clinical background. The resources for which no consensus was reached in the questionnaire were discussed, and after discussion participants were asked to vote on accepting or rejecting the resources using the polls function in Teams. A 70% threshold for acceptance of resources was used. A summary of the results was shared with the participants on completion of the consensus procedure. This procedure was held for the two expert panels mentioned above: first, a consensus procedure with PHEs (27 January and 14 February 2022; two sessions needed), and a separate consensus procedure with clinicians (30 May 2022). The panel of PHEs included participants from Austria, Finland, Germany, Ireland, Italy, the Netherlands, Portugal, Romania and Sweden; the panel of clinicians included participants from Finland, Ireland, the Netherlands and Portugal.

### Ethics and data protection

Respondents were asked to give informed consent to participate in the questionnaire and the consensus procedure. Respondents participating in the consensus procedure were also asked to consent to recording the meeting for reviewing purposes and informed that no part of the recording would be published in any shape or form. All data were pseudonymised for processing and analysis anonymously. Questionnaire data are stored at the National Institute for Public Health and the Environment on a secure drive and can only be accessed by the researchers involved in this study. Approval from a medical ethics committee was deemed unnecessary for this study by the Centre for Clinical Expertise of the National Institute for Public Health and the Environment (LCI-527).

### Patient and public involvement

Patients or members of the public were not involved in the design, conduct, reporting or dissemination of this study.

## Results

### Respondents

We held two separate consensus procedures: one with 17 PHEs (Delphi 1), which was split into two sessions due to time constraints, and one with 16 clinicians (Delphi 2). For Delphi 1, 17 PHEs participated in the questionnaire, of which 10 participated in the first session of the online consensus procedure 7 in the second session. PHEs were mainly active within public health organisations (n=9), followed by government agencies (n=3), non-governmental organisations and research institutes or universities (n=2 each). One participant was active in a communications company that had provided pandemic-related communication services. For Delphi 2, 16 clinicians participated in the questionnaire, of which 7 participated in the online consensus procedure. Clinicians worked mainly in hospitals (n=7), followed by public health organisations (n=5), research institutes or universities (n=3) and general practice (n=1). The full characteristics of the participants are given in table 1.

**Table 1:** Characteristics of participants in this study

Type of organisation	Public health experts			Clinicians	
	Questionnaire (n=17)	Consensus meeting 1 (n=10)	Consensus meeting 2 (n=7)	Questionnaire (n=16)	Consensus meeting (n=7)
Public health organisation	9	2	2	5	
Non-governmental organisation	2	4	2		
Government agency	3	3	2		
Research institute/university	2	1	1	3	2
Communications company	1				
Hospital				7	5
General practice				1	
Years’ experience	3 (median)6.6 (average)	3 (median)3.9 (average)	3 (median)3 (average)	6.5 (median)8.2 (average)	3 (median)7.1 (average)
Country of employment					
Austria	1	3	2		
Finland	1	1	1	7	2
Germany	2	1	1		
Ireland	3	3	3	1	
Italy	2	1			
Netherlands	3			6	5
Portugal	1	1		2	
Romania	3				
Sweden	1				

### Results of Delphi 1 consensus procedure with PHEs

Results from the questionnaire for PHEs participating in Delphi 1 (online supplemental material 3) were that for pandemic preparedness planning: 35/53 (66%) resources were accepted, 11/53 (21%) were rejected and 7/53 (13%) required discussion in the consensus meeting. For pandemic modelling, after the questionnaire 34/53 (64%) resources were accepted, 11/53 (21%) were rejected and 8/53 (15%) required further discussion. During the Delphi 1 consensus meeting, PHEs accepted four and rejected three more resources for pandemic preparedness planning, and accepted five and rejected three more resources for pandemic modelling. Five new resources were suggested in the questionnaire; of these five, all were accepted for pandemic preparedness planning, but only two were accepted for pandemic modelling (table 2).

**Table 2:** Outcomes of the consensus discussion on resources that were added by participants in the questionnaire

	Pandemic preparedness planning	Resource modelling
Suggested resources: public health
PPE for mobile teams	Accepted	Rejected
Guidelines/SOPs on prevention, notification, interventions, coordination within Europe and on patient management, PPE use, treatment guidelines, etc	Accepted	Rejected
Shelters/quarantine facilities for refugees/homeless	Accepted	Rejected
Delivery of new PPE, equipment and reagents (procurement)	Accepted	Accepted
Number of ambulance services for suspected cases	Accepted	Accepted
Suggested resources: clinicians		
Primary care PPE	Accepted	Accepted
Primary care beds	Accepted	Accepted
Primary care physicians/GPs	Accepted	Accepted
Hand disinfectants	Accepted	Accepted
Medication for mechanical ventilation	Accepted	Accepted
Disposables for mechanical ventilation and intravenous treatment	Accepted	Accepted
Post-pandemic staff to address postponed care	Rejected	Rejected

The upper section of the table concerns suggestions made by, and discussed by public health experts. The bottom section concerns suggestions made by, and discussed by clinicians. Green indicates included and, pink indicates rejected. PPE: ; SOP: standard operating procedure; GP: general practitioner; : intravenous.

GPgeneral practitionerPPEpersonal protective equipmentSOPstandard operating procedure

### Results of Delphi 2 consensus procedure with clinicians

Results from the questionnaire for clinicians participating in Delphi 2 (online supplemental material 4) were that for pandemic preparedness planning: 41/53 (77%) resources were accepted, 10/53 (19%) were rejected and 2/53 (4%) required discussion in the consensus meeting. For pandemic modelling, after the questionnaire 39/53 (74%) resources were accepted, 9/53 (17%) were rejected and 5/53 (9%) required further discussion. During the Delphi 2 consensus meeting, clinicians accepted one and rejected one more resource for pandemic preparedness planning, and accepted three and rejected two more resources for pandemic modelling. Seven new resources were suggested in the questionnaire; of these, six were accepted for both pandemic preparedness planning and pandemic modelling (table 2).

### Differences between panels

As shown in the results above, more discussion was required between PHEs than clinicians (15 and 7 resources, respectively). Clinicians also included three more resources than PHEs in the final results. In total, PHEs and clinicians had the same outcome for both pandemic preparedness planning and pandemic modelling for 40 resources, excluding new suggestions. Of these 40, 33 were accepted and 7 were rejected (table 3). There was a mixed outcome for 13 resources, with consensus between panels for either one of the purposes for some resources, or consensus within a panel for both purposes but not between panels (table 4).

**Table 3:** Overview of resources that were included or rejected by both panels for both pandemic preparedness planning and pandemic resource modelling after the consensus rounds

Material resources	Human resources	Pharmaceutical resources
Duration of mechanical ventilation use	Ambulance arrival time	Prophylactic antibiotics dose
Home care rehabilitation oxygen	Contact tracing rate	Prophylactic antivirals dose
Hospital PPE stock	Contact tracing staff	Prophylactic antivirals efficacy
ICU admission rate	Home care nursing staff	Therapeutic antibiotics dose
ICU length of stay	Home care rehabilitation physiotherapy	Therapeutic antivirals efficacy
ICU length of stay with mechanical ventilation	ICU nurse absenteeism	Vaccination administration speed
Non-ICU admission rate	ICU physician absenteeism	Vaccine dose
Non-ICU length of stay	Increased ambulance demand	Vaccine efficacy
PPE kit stockpile	Laboratory testing capacity	Vaccine manufacturing capacity
PPE usage per bed per day	Nurses per ICU bed	
Rate of mechanical ventilation use	Nurses per non-ICU bed	
Test specificity	Physicians per ICU bed	
Time to test result	Testing facility capacity	
Total ICU beds available	Testing facility staff	
Total non-ICU beds available		
Total public health testing capacity		
X-ray/radiography increased demand		

Green indicates included and pink indicates rejected.

ICU, intensive care unit; PPE, personal protective equipment

**Table 4:** Overview of resources for which consensus was reached between public health experts and clinicians

	Pandemic preparedness planning		Resource modelling	
	Public health experts	Clinicians	Public health experts	Clinicians
Material resources
Testing reagents				
Test sensitivity				
Conversion rate non-ICU to ICU				
Oxygen demand per bed				
Home care PPE stock				
Home care PPE usage				
Human resources				
Physicians per non-ICU bed				
Non-ICU physician absenteeism				
Non-ICU nurse absenteeism				
Home care rehabilitation nursing staff				
Pharmaceutical resources				
Therapeutic antivirals dose				
Prophylactic antibiotics efficacy				
Therapeutic antibiotics efficacy				

Green indicates included and pink indicates rejected.

ICUintensive care unitPPEpersonal protective equipment

## Discussion

The two international consensus procedures with two panels of public health and clinical experts resulted in a core set of 33 resources (15 material resources, 12 human resources, 6 pharmaceutical resources) considered very relevant for both pandemic preparedness planning and pandemic resource modelling by PHEs and clinicians. Seven resources were rejected by both panels, of which four were pharmaceutical resources. No consensus was reached on 13 resources, notably regarding home care and pharmaceutical resources. Clinicians accepted three more resources in total compared with the PHEs, but both panels accepted and rejected an equal number of resources for both pandemic preparedness planning and pandemic resource modelling. A number of rejections were highly unexpected in light of the experiences with COVID-19. With the demand for intensive care unit (ICU) beds during COVID-19 being the most important indicator in a number of countries to monitor the progression of the pandemic and formulate countermeasures, the conversion rate from non-ICU to ICU beds was expected to be important for pandemic preparedness planning. However, the panel of clinicians rejected this resource for pandemic preparedness planning. Ventilator demand was also high during COVID-19, which in turn caused a high demand for oxygen. Oxygen shortages have been reported, especially in low- and middle-income countries.^20^ Notably though, clinicians rejected the resources ‘conversion rate non-ICU to ICU’ and ‘oxygen demand per bed’ for pandemic preparedness planning, while these were accepted by the PHEs and accepted by both for pandemic resource modelling. The COVID-19 pandemic also saw patients receiving care at home to recover after a hospital stay, an important type of care delivery to enable quicker discharge from the hospital and thus increase bed availability. The importance of this was reflected by clinicians including resources for home care. PHEs, however, did not include these resources, possibly due to lower visibility of this type of care delivery.

Differences between PHEs and clinicians were also observed in the resources they suggested in addition: PHEs suggested resources all relevant to the public health domain while clinicians suggested resources relevant to clinical practice, both in primary care and hospital care. This highlights the differences in perspective between PHEs and clinicians and emphasises the importance of including experts from varied backgrounds when conducting pandemic preparedness activities. If this is not done, preparedness activities will risk having blind spots or leading to fragmented responses. A secondary benefit of including experts from varied backgrounds in preparedness activities is that it connects institutions and healthcare professionals from public health, hospital care, primary care and other levels of care, strengthening formal and informal networks and allowing for a better understanding of each other’s perspective. Regarding resources, the importance of various resources for preparedness may be misjudged, or resource gaps may be missed when the professionals involved are not a diverse group. This study provides added value by presenting a core set of resources that should always be considered for pandemic preparedness planning and pandemic resources modelling for emerging viral respiratory diseases.

While much research has been done on hospital resource allocation during pandemics,^21^,^25^ and on hospital capacity planning in non-pandemic situations,^26^ to our knowledge few studies have been published using expert opinion to determine which resources are most important for pandemic preparedness. Bayram *et al* included a pandemic influenza scenario in their Delphi study and evaluated 132 hospital resources, with 58 being considered critical by >50% of participants. Similar resources in our study and the study of Bayram *et al*^15^ were PPE, hand sanitiser, physicians, nurses, mechanical ventilation, ICU beds, antibiotics and radiography, all included by the panel in the study of Bayram *et al*. Direct comparison between both Delphi studies is difficult, however, due to different operational definitions of resources. The study by Bayram *et al* also included many more clinical care-specific resources and did not include resources related to public health activities. We found no other work investigating both public health and healthcare resources. Further, we did not include financial resources in this study. The cost of procurement and stockpiling of resources is an important aspect of policy and decision-making on resources but it is beyond the scope of this work.

### Strengths and limitations

Our Delphi study was conducted with two separate international panels. Our initial intentions were to conduct one Delphi with a mixed panel of both PHEs and clinicians; however, this was adapted as our initial recruitment yielded only PHEs. A probable cause for the initial failure to recruit clinicians lies in the timing: recruitment was started in October 2021, just before the Omicron variant emerged and resulted in a large COVID-19 wave in Europe. The resulting increased hospitalisations and clinical load may have prevented clinicians from participating in our study. Sufficient PHEs were recruited to start a consensus round, and in the end of 2022, when the pressure of COVID-19 cases was reduced, a panel of clinicians was recruited for the second consensus round. It would have been valuable to have these two groups of experts together in one panel to facilitate understanding between the panels and gain more insight into the reasoning behind the resources for which there was a discrepancy between the two groups of experts. Because of the sequential order of both Delphi studies, additional resources that were suggested by panel members in the Delphi 2 could not be discussed by the panel of Delphi 1. Nonetheless, we felt it very important to include both perspectives in this study in order to provide a complete picture. As a whole, the combination of literature review and the Delphi process was a useful method to obtain expert opinion using a structured process.

Bias may be introduced through the composition of the panel of experts where certain backgrounds or areas of expertise can influence the outcomes of the questionnaire, and more outspoken participants may influence the online discussions.^16^ To address this, the panel moderators explicitly invited all experts to speak out during the online rounds. In our study, participants in both panels represented a variety of areas of expertise. Both panels did not have the exact same composition with regard to the country of employment, and the panel of clinicians featured higher representation from the Netherlands and Finland. This may be a limitation with regard to the comparison of outcomes and result in a possible bias toward outcomes relevant to those countries. No country was over-represented in the panel of PHEs, thus reducing the risk of bias in that regard. However, differences in views may also occur within a country, and we believe that some of the unexpected findings are more due to differences in professional background and perspective between PHEs and clinicians rather than different countries of origin. There was a high degree of consensus between the two expert panels, and the set of resources that both panels agree on is valuable as it is supported by experts from varied European backgrounds and thus more applicable to other European countries. Regarding the online discussions, we tried to reduce bias or skewing of the discussion through moderation and the use of a blind, online voting system which proved to be easily implemented and user-friendly.

### Conclusion and recommendations

This study identified a core set of 33 resources that PHEs and clinicians consider essential for pandemic preparedness planning and resource modelling for emerging viral respiratory diseases. Our study has demonstrated the core components of resource modelling required for pandemic preparedness planning to ensure an effective response to future threats. The results of this study have also been applied to the work on developing a resource modelling tool in the PANDEM-2 project. The high level of agreement between both panels indicated a shared view on pandemic preparedness. The disagreement on a small part of resources indicates the importance of including experts of complementary backgrounds when conducting preparedness activities. These findings are highly relevant for public health professionals and healthcare managers, but also for researchers who can use these findings when modelling the impact of outbreaks on resource availability in regions.

## supplementary material

10.1136/bmjopen-2023-079609 online supplemental file 1

## Data Availability

All data relevant to the study are included in the article or uploaded as supplementary information.
