# Supplementary material for: Identifying essential resource parameters for pandemic preparedness and response: an international Delphi study within the EU PANDEM-2 project
Source: BMJ Open. 2024 Dec 15;14(12):e079609. doi: 10.1136/bmjopen-2023-079609 (PMC11647290; doi:10.1136/bmjopen-2023-079609)
Supplement: online supplemental file 1 [file bmjopen-14-12-s001.docx]

Identifying essential resources for pandemic response: an international Delphi study within the EU-PANDEM-2 project

Supplementary material

Supplementary material 1: ACCORD reporting guidelines

| Item No. | Section | Checklist Item (*help text*) | Page No. |
| --- | --- | --- | --- |
| T1 | **Title** | Identify the article as reporting a consensus exercise and state the consensus methods used in the title.  *For example, Delphi or nominal group technique.* | 1 |
| I1 | **Introduction** | Explain why a consensus exercise was chosen over other approaches. | 4 |
| I2 |  | State the aim of the consensus exercise, including its intended audience and geographical scope (national, regional, global). | 4, 5 |
| I3 |  | If the consensus exercise is an update of an existing document, state why an update is needed, and provide the citation for the original document. | n/a |
| M1 | **Methods**  Registration | If the study or study protocol was prospectively registered, state the registration platform and provide a link. If the exercise was not registered, this should be stated.  *Recommended to include the date of registration.* | 5 |
| M2 | Selection of SC and/or panellists | Describe the role(s) and areas of expertise or experience of those directing the consensus exercise.  *For example, whether the project was led by a chair, co-chairs or a steering committee, and, if so, how they were chosen. List their names if appropriate, and whether there were any subgroups for individual steps in the process.* | 8 |
| M3 |  | Explain the criteria for panellist inclusion and the rationale for panellist numbers. State who was responsible for panellist selection. | 6 |
| M4 |  | Describe the recruitment process (how panellists were invited to participate).  *Include communication/advertisement method(s) and locations, numbers of invitations sent, and whether there was centralised oversight of invitations or if panellists were asked/allowed to suggest other members of the panel.* | 5, 6 |
| M5 |  | Describe the role of any members of the public, patients or carers in the different steps of the study. | 8 |
| M6 | Preparatory research | Describe how information was obtained prior to generating items or other materials used during the consensus exercise.  *This might include a literature review, interviews, surveys, or another process.* | 5, review published separately |
| M7 |  | Describe any systematic literature search in detail, including the search strategy and dates of search or the citation if published already.  *Provide the details suggested by the reporting guideline PRISMA and the related PRISMA-Search extension.* | review published separately |
| M8 |  | Describe how any existing scientific evidence was summarised and if this evidence was provided to the panellists. | review published separately |
| M9 | Assessing consensus | Describe the methods used and steps taken to gather panellist input and reach consensus (for example, Delphi, RAND-UCLA, nominal group technique).  *If modifications were made to the method in its original form, provide a detailed explanation of how the method was adjusted and why this was necessary for the purpose of your consensus-based study.* | 7, 8 |
| M10 |  | Describe how each question or statement was presented and the response options. State whether panellists were able to or required to explain their responses, and whether they could propose new items.  *Where possible, present the questionnaire or list of statements as supplementary material.* | 6, suppl. 2 |
| M11 |  | State the objective of each consensus step.  *A step could be a consensus meeting, a discussion or interview session, or a Delphi round.* | 7, 8 |
| M12 |  | State the definition of consensus (for example, number, percentage, or categorical rating, such as ‘agree’ or ‘strongly agree’) and explain the rationale for that definition. | 6 |
| M13 |  | State whether items that met the prespecified definition of consensus were included in any subsequent voting rounds. | 7, 8 |
| M14 |  | For each step, describe how responses were collected, and whether responses were collected in a group setting or individually. | 7, 8 |
| M15 |  | Describe how responses were processed and/or synthesised.  *Include qualitative analyses of free-text responses (for example, thematic, content or cluster analysis) and/or quantitative analytical methods, if used.* | 7, 8 |
| M16 |  | Describe any piloting of the study materials and/or survey instruments.  *Include how many individuals piloted the study materials, the rationale for the selection of those individuals, any changes made as a result and whether their responses were used in the calculation of the final consensus. If no pilot was conducted, this should be stated.* | 6 |
| M17 |  | If applicable, describe how feedback was provided to panellists at the end of each consensus step or meeting.  *State whether feedback was quantitative (for example, approval rates per topic/item) and/or qualitative (for example, comments, or lists of approved items), and whether it was anonymised.* | 7, 8 |
| M18 |  | State whether anonymity was planned in the study design. Explain where and to whom it was applied and what methods were used to guarantee anonymity. | 7, 8 |
| M19 |  | State if the steering committee was involved in the decisions made by the consensus panel.  *For example, whether the steering committee or those managing consensus also had voting rights.* | n/a |
| M20 | Participation | Describe any incentives used to encourage responses or participation in the consensus process.  *For example, were invitations to participate reiterated, or were participants reimbursed for their time.* | n/a |
| M21 |  | Describe any adaptations to make the surveys/meetings more accessible.  *For example, the languages in which the surveys/meetings were conducted and whether translations or plain language summaries were available*. | n/a |
| R1 | Results | State when the consensus exercise was conducted. List the date of initiation and the time taken to complete each consensus step, analysis, and any extensions or delays in the analysis. | 8 |
| R2 |  | Explain any deviations from the study protocol, and why these were necessary.  *For example, addition of panel members during the exercise, number of consensus steps, stopping criteria; report the step(s) in which this occurred.* | 9 |
| R3 |  | For each step, report quantitative (number of panellists, response rate) and qualitative (relevant socio-demographics) data to describe the participating panellists. | 9, 10 |
| R4 |  | Report the final outcome of the consensus process as qualitative (for example, aggregated themes from comments) and/or quantitative (for example, summary statistics, score means, medians and/or ranges) data. | 10-13 |
| R5 |  | List any items or topics that were modified or removed during the consensus process. Include why and when in the process they were modified or removed. | n/a |
| D1 | Discussion | Discuss the methodological strengths and limitations of the consensus exercise.  *Include factors that may have impacted the decisions (for example, response rates, representativeness of the panel, potential for feedback during consensus to bias responses, potential impact of any non-anonymised interactions).* | 15, 16 |
| D2 |  | Discuss whether the recommendations are consistent with any pre-existing literature and, if not, propose reasons why this process may have arrived at alternative conclusions. | 13-15 |
| O1 | Other information | List any endorsing organisations involved and their role. | 17 |
| O2 |  | State any potential conflicts of interests, including among those directing the consensus study and panellists. Describe how conflicts of interest were managed. | 17 |
| O3 |  | State any funding received and the role of the funder.  *Specify, for example, any funder involvement in the study concept/design, participation in the steering committee, conducting the consensus process, funding of any medical writing support. This could be disclosed in the methods or in the relevant transparency section of the manuscript. Where a funder did not play a role in the process or influence the decisions reached, this should be specified.* | 17 |

Supplementary material 2: Delphi questionnaire

Delphi study on resources for pandemic planning and response: development of a resource model for the PANDEM-2 dashboard

Introduction

This questionnaire is part of a Delphi study on resources for pandemic planning and response. For more information on the context of this study, please refer to the information letter accompanying this questionnaire. This study is being conducted as part of work package 4 of the PANDEM-2 project. This work package is coordinated by the National University of Ireland, Galway (NUIG); this study is executed by the National Institute for Public Health and the Environment (RIVM), and Radboudumc from the Netherlands.

To determine which resources are most relevant for pandemic preparedness and response and subsequent resource modeling, we use the Delphi process. The Delphi process consists of three steps:
step 1: literature review,
step 2: written questionnaire and
step 3: consensus discussion with international experts.

This method allows us to use both knowledge from literature and expert opinion to arrive at a relevant and well-defined set of resources which will serve as input variables for the model. To narrow the scope: we create this model for the specific purpose of simulating resource use in a pandemic with a viral respiratory illness, such as SARS or COVID-19.

This questionnaire consists of three parts:
(a) some information about yourself as professional
(b) ranking relevance of resources for **pandemic planning and response**. Resource were derived from a literature review and the AsiaFluCap project. Resources are categorized by group.
(c) ranking relevance of resources for **a pandemic resource model**. The same group of resources is used as in step B.

Part B and C consist of the same set of resource variables, but the purpose to which you rank them is different. Please keep this in mind.

This questionnaire is targeted to professionals working in pandemic planning and response, either at government institutes, hospitals or local/regional/national/international public health organisations. Minimum experience of three years is preferred. **Please share this questionnaire with relevant colleagues inside or outside your organisation.**
This questionnaire will take about 15-20 minutes to complete.

DATA PROTECTION: All personal information will be stored securely and will be used only for the purpose described above. The raw data will be stored at the RIVM in an internal institutional database for a duration of 10 years, where only the work package team have access and that has proper IT protection, and will not be transferred outside the organizational structure. Raw data will be pseudonymized for analysis and publication of results. No personally identifiable information will be published.No personal information will be included in the publication of the results of the survey. You can request removal from our records at any time by emailing *berend.beishuizen@rivm.nl*

It is possible to save your response to the questionnaire and restart at a later moment.

I agree to participate in this questionnaire □

| Part A. Demographic information |  |  |  |  |  |  |  |  |  |  |  |
| --- | --- | --- | --- | --- | --- | --- | --- | --- | --- | --- | --- |
|  |  |  |  |  |  |  |  |  |  |  |  |
| Which country do you represent? | <select country> | | | | |  |  |  |  |  |  |
|  | Other: | |  |  |  |  |  |  |  |  |  |
| For which type of organisation do you work? | Research institute | | | | | |  |  |  |  |  |
|  | Government agency | | | | | |  |  |  |  |  |
|  | Public health organzation | | | | | | | |  |  |  |
|  | Hospital | | |  |  |  |  |  |  |  |  |
|  | Other: | |  |  |  |  |  |  |  |  |  |
| What is your position at your organization? |  |  |  |  |  |  |  |  |  |  |  |
| How many years of experience do you have in pandemic planning? | <select years> | | | |  |  |  |  |  |  |  |
| E-mail adress where we can contact you for the consensus meeting | | | | |  |  |  |  |  |  |  |
|  |  |  |  |  |  |  |  |  |  |  |  |
| Part B. |  |  |  |  |  |  |  |  |  |  |  |
| Instructions, please read carefully |  |  |  |  |  |  |  |  |  |  |  |
| This section is focused on the relevance of resources for pandemic planning and response activities (including training exercises) in the context of an outbreak with a respiratory infection. For each resource variable, please rank the relevance for pandemic planning and response on a scale of 1 to 9, 1 being ‘not relevant at all’ and 9 being ‘extremely relevant’. An opportunity is given at the end of each section to provide open-text comments or to add resource variables which were not yet included but which you consider relevant for that section.  It is possible to save your response to the questionnaire and restart at a later moment. | | | | | | | | | | |  |
|  |  |  |  |  |  |  |  |  |  |  |  |
|  |  |  |  |  |  |  |  |  |  |  |  |
|  |  |  |  |  |  |  |  |  |  |  |  |
|  |  |  |  |  |  |  |  |  |  |  |  |
|  |  |  |  |  |  |  |  |  |  |  |  |
|  |  |  |  |  |  |  |  |  |  |  |  |
|  |  |  |  |  |  |  |  |  |  |  |  |
|  |  |  |  |  |  |  |  |  |  |  |  |
|  |  |  |  |  |  |  |  |  |  |  |  |
|  |  |  |  |  |  |  |  |  |  |  |  |
| **Material resources for pandemic planning and response** | |  |  |  |  |  |  |  |  |  |  |
|  | Not relevant at all | | | | | |  |  | Extremely relevant | |  |
| Public Health | 1 | 2 | 3 | 4 | 5 | 6 | 7 | 8 | 9 | I don't know |  |
| Total public health testing capacity |  |  |  |  |  |  |  |  |  |  |  |
| Testing reagents |  |  |  |  |  |  |  |  |  |  |  |
| Test sensitivity |  |  |  |  |  |  |  |  |  |  |  |
| Test specificity |  |  |  |  |  |  |  |  |  |  |  |
| Time to test result |  |  |  |  |  |  |  |  |  |  |  |
|  | Not relevant at all | | | | | |  |  | Extremely relevant | |  |
| General hospital | 1 | 2 | 3 | 4 | 5 | 6 | 7 | 8 | 9 | I don't know |  |
| Hospital PPE stock |  |  |  |  |  |  |  |  |  |  |  |
| PPE usage per bed per day |  |  |  |  |  |  |  |  |  |  |  |
| PPE kit stockpile |  |  |  |  |  |  |  |  |  |  |  |
| Conversion rate non-ICU to ICU |  |  |  |  |  |  |  |  |  |  |  |
| Oxygen demand per bed |  |  |  |  |  |  |  |  |  |  |  |
| Rate of mechanical ventilation use |  |  |  |  |  |  |  |  |  |  |  |
| X-ray/radiography increased demand |  |  |  |  |  |  |  |  |  |  |  |
|  | Not relevant at all | | | | | |  |  | Extremely relevant | |  |
|  | 1 | 2 | 3 | 4 | 5 | 6 | 7 | 8 | 9 | I don't know |  |
| Non-ICU |  |  |  |  |  |  |  |  |  |  |  |
| Total non-ICU beds available |  |  |  |  |  |  |  |  |  |  |  |
| Non-ICU admission rate |  |  |  |  |  |  |  |  |  |  |  |
| Non-ICU length of stay |  |  |  |  |  |  |  |  |  |  |  |
|  | Not relevant at all | | | | | |  |  | Extremely relevant | |  |
| ICU | 1 | 2 | 3 | 4 | 5 | 6 | 7 | 8 | 9 | I don't know |  |
| Total ICU beds available |  |  |  |  |  |  |  |  |  |  |  |
| ICU admission rate |  |  |  |  |  |  |  |  |  |  |  |
| ICU length of stay |  |  |  |  |  |  |  |  |  |  |  |
| ICU length of stay with mechanical ventilation |  |  |  |  |  |  |  |  |  |  |  |
|  | Not relevant at all | | | | | |  |  | Extremely relevant | |  |
| Home care | 1 | 2 | 3 | 4 | 5 | 6 | 7 | 8 | 9 | I don't know |  |
| Home care PPE stock |  |  |  |  |  |  |  |  |  |  |  |
| Home care PPE usage |  |  |  |  |  |  |  |  |  |  |  |
| Home care rehabilitation oxygen |  |  |  |  |  |  |  |  |  |  |  |
|  |  |  |  |  |  |  |  |  |  |  |  |
| If you have any questions or remarks about the resources or resource variables above, please use the box below. | | | | | | | | | | |  |
|  |  |  |  |  |  |  |  |  |  |  |  |
|  |  |  |  |  |  |  |  |  |  |  |  |
| If you would like to add resources or resource variables to the Material resources section, please use the box below. | | | | | | | | | | |  |
|  |  |  |  |  |  |  |  |  |  |  |  |
|  |  |  |  |  |  |  |  |  |  |  |  |
|  |  |  |  |  |  |  |  |  |  |  |  |
| **Human resources for pandemic planning and response** | |  |  |  |  |  |  |  |  |  |  |
|  | Not relevant at all | | | | | |  |  | Extremely relevant | |  |
| Public Health | 1 | 2 | 3 | 4 | 5 | 6 | 7 | 8 | 9 | I don't know |  |
| Testing facility staff |  |  |  |  |  |  |  |  |  |  |  |
| Testing facility capacity |  |  |  |  |  |  |  |  |  |  |  |
| Contact tracing staff |  |  |  |  |  |  |  |  |  |  |  |
| Contact tracing rate |  |  |  |  |  |  |  |  |  |  |  |
|  | Not relevant at all | | | | | |  |  | Extremely relevant | |  |
| General hospital | 1 | 2 | 3 | 4 | 5 | 6 | 7 | 8 | 9 | I don't know |  |
| Laboratory testing capacity |  |  |  |  |  |  |  |  |  |  |  |
| Ambulance arrival time |  |  |  |  |  |  |  |  |  |  |  |
| Increase ambulance demand |  |  |  |  |  |  |  |  |  |  |  |
|  | Not relevant at all | | | | | |  |  | Extremely relevant | |  |
| Non-ICU | 1 | 2 | 3 | 4 | 5 | 6 | 7 | 8 | 9 | I don't know |  |
| Physicians per non-ICU bed |  |  |  |  |  |  |  |  |  |  |  |
| Nurses per non-ICU bed |  |  |  |  |  |  |  |  |  |  |  |
| Non-ICU physician absenteeism |  |  |  |  |  |  |  |  |  |  |  |
| Non-ICU nurse absenteeism |  |  |  |  |  |  |  |  |  |  |  |
|  | Not relevant at all | | | | | |  |  | Extremely relevant | |  |
| ICU | 1 | 2 | 3 | 4 | 5 | 6 | 7 | 8 | 9 | I don't know |  |
| Physicians per ICU bed |  |  |  |  |  |  |  |  |  |  |  |
| Nurses per ICU bed |  |  |  |  |  |  |  |  |  |  |  |
| ICU physician absenteeism |  |  |  |  |  |  |  |  |  |  |  |
| ICU nurse absenteeism |  |  |  |  |  |  |  |  |  |  |  |
|  |  |  |  |  |  |  |  |  |  |  |  |
| If you have any questions or remarks about the resources or resource variables above, please use the box below. | | | | | | | | | | |  |
|  |  |  |  |  |  |  |  |  |  |  |  |
|  |  |  |  |  |  |  |  |  |  |  |  |
| If you would like to add resources or resource variables to the Human resources section, please use the box below. | | | | | | | | | | |  |
|  |  |  |  |  |  |  |  |  |  |  |  |
|  |  |  |  |  |  |  |  |  |  |  |  |
|  |  |  |  |  |  |  |  |  |  |  |  |
| **Therapeutics for pandemic planning and response** | Not relevant at all | | | | | |  |  | Extremely relevant | |  |
|  | 1 | 2 | 3 | 4 | 5 | 6 | 7 | 8 | 9 | I don't know |  |
| Vaccine dose |  |  |  |  |  |  |  |  |  |  |  |
| Vaccine administration speed |  |  |  |  |  |  |  |  |  |  |  |
| Vaccine manufacturing capacity |  |  |  |  |  |  |  |  |  |  |  |
| Vaccine efficacy |  |  |  |  |  |  |  |  |  |  |  |
| Prophylactic antivirals dose |  |  |  |  |  |  |  |  |  |  |  |
| Prophylactic antivirals efficacy |  |  |  |  |  |  |  |  |  |  |  |
| Therapeutic antivirals dose |  |  |  |  |  |  |  |  |  |  |  |
| Therapeutic antivirals efficacy |  |  |  |  |  |  |  |  |  |  |  |
| Prophylactic antibiotics dose |  |  |  |  |  |  |  |  |  |  |  |
| Prophylactic antibiotics efficacy |  |  |  |  |  |  |  |  |  |  |  |
| Therapeutic antibiotics dose |  |  |  |  |  |  |  |  |  |  |  |
| Therapeutic antibiotics efficacy |  |  |  |  |  |  |  |  |  |  |  |
|  |  |  |  |  |  |  |  |  |  |  |  |
| If you have any questions or remarks about the resources or resource variables above, please use the box below. | | | | | | | | | | |  |
|  |  |  |  |  |  |  |  |  |  |  |  |
|  |  |  |  |  |  |  |  |  |  |  |  |
| If you would like to add resources or resource variables to the Therapeutics resources section, please use the box below. | | | | | | | | | | |  |
|  |  |  |  |  |  |  |  |  |  |  |  |
|  |  |  |  |  |  |  |  |  |  |  |  |
|  |  |  |  |  |  |  |  |  |  |  |  |
| Part C. |  |  |  |  |  |  |  |  |  |  |  |
| Instructions, please read carefully |  |  |  |  |  |  |  |  |  |  |  |
| This section is focused on the relevance of resources for a pandemic resource model. As part of the PANDEM-2 project, we are developing a resource model which is based on a SEIR model enhanced with contact tracing, testing, vaccination and also post-hospital care of recovering patients. The purpose of this model is to allow public health professionals such as policy advisors or pandemic planners to model the impact of an infectious disease on resources in the healthcare system, or conversely to model output variables such as morbidity and mortality based on given resource constraints. This model is developed for the scenario of an outbreak with a respiratory infection.  For each resource variable, please rank the relevance for a pandemic resource model on a scale of 1 to 9, 1 being ‘not relevant at all’ and 9 being ‘extremely relevant’. An opportunity is given at the end of each section to provide open-text comments or to add resource variables which were not yet included but which you consider relevant for that section.  It is possible to save your response to the questionnaire and restart at a later moment. | | | | | | | | | | |  |
|  |  |  |  |  |  |  |  |  |  |  |  |
|  |  |  |  |  |  |  |  |  |  |  |  |
|  |  |  |  |  |  |  |  |  |  |  |  |
|  |  |  |  |  |  |  |  |  |  |  |  |
|  |  |  |  |  |  |  |  |  |  |  |  |
|  |  |  |  |  |  |  |  |  |  |  |  |
|  |  |  |  |  |  |  |  |  |  |  |  |
|  |  |  |  |  |  |  |  |  |  |  |  |
|  |  |  |  |  |  |  |  |  |  |  |  |
|  |  |  |  |  |  |  |  |  |  |  |  |
|  |  |  |  |  |  |  |  |  |  |  |  |
|  |  |  |  |  |  |  |  |  |  |  |  |
|  |  |  |  |  |  |  |  |  |  |  |  |
|  |  |  |  |  |  |  |  |  |  |  |  |
|  |  |  |  |  |  |  |  |  |  |  |  |
| **Material resources for a pandemic resource model** |  |  |  |  |  |  |  |  |  |  |  |
|  | Not relevant at all | | | | | |  |  | Extremely relevant | |  |
| Public Health | 1 | 2 | 3 | 4 | 5 | 6 | 7 | 8 | 9 | I don't know |  |
| Total public health testing capacity |  |  |  |  |  |  |  |  |  |  |  |
| Testing reagents |  |  |  |  |  |  |  |  |  |  |  |
| Test sensitivity |  |  |  |  |  |  |  |  |  |  |  |
| Test specificity |  |  |  |  |  |  |  |  |  |  |  |
| Time to test result |  |  |  |  |  |  |  |  |  |  |  |
|  | Not relevant at all | | | | | |  |  | Extremely relevant | |  |
| General hospital | 1 | 2 | 3 | 4 | 5 | 6 | 7 | 8 | 9 | I don't know |  |
| Hospital PPE stock |  |  |  |  |  |  |  |  |  |  |  |
| PPE usage per bed per day |  |  |  |  |  |  |  |  |  |  |  |
| PPE kit stockpile |  |  |  |  |  |  |  |  |  |  |  |
| Conversion rate non-ICU to ICU |  |  |  |  |  |  |  |  |  |  |  |
| Oxygen demand per bed |  |  |  |  |  |  |  |  |  |  |  |
| Rate of mechanical ventilation use |  |  |  |  |  |  |  |  |  |  |  |
| X-ray/radiography increased demand |  |  |  |  |  |  |  |  |  |  |  |
|  | Not relevant at all | | | | | |  |  | Extremely relevant | |  |
|  | 1 | 2 | 3 | 4 | 5 | 6 | 7 | 8 | 9 | I don't know |  |
| Non-ICU |  |  |  |  |  |  |  |  |  |  |  |
| Total non-ICU beds available |  |  |  |  |  |  |  |  |  |  |  |
| Non-ICU admission rate |  |  |  |  |  |  |  |  |  |  |  |
| Non-ICU length of stay |  |  |  |  |  |  |  |  |  |  |  |
|  | Not relevant at all | | | | | |  |  | Extremely relevant | |  |
| ICU | 1 | 2 | 3 | 4 | 5 | 6 | 7 | 8 | 9 | I don't know |  |
| Total ICU beds available |  |  |  |  |  |  |  |  |  |  |  |
| ICU admission rate |  |  |  |  |  |  |  |  |  |  |  |
| ICU length of stay |  |  |  |  |  |  |  |  |  |  |  |
| ICU length of stay with mechanical ventilation |  |  |  |  |  |  |  |  |  |  |  |
|  | Not relevant at all | | | | | |  |  | Extremely relevant | |  |
| Home care | 1 | 2 | 3 | 4 | 5 | 6 | 7 | 8 | 9 | I don't know |  |
| Home care PPE stock |  |  |  |  |  |  |  |  |  |  |  |
| Home care PPE usage |  |  |  |  |  |  |  |  |  |  |  |
| Home care rehabilitation oxygen |  |  |  |  |  |  |  |  |  |  |  |
|  |  |  |  |  |  |  |  |  |  |  |  |
| If you have any questions or remarks about the resources or resource variables above, please use the box below. | | | | | | | | | | |  |
|  |  |  |  |  |  |  |  |  |  |  |  |
|  |  |  |  |  |  |  |  |  |  |  |  |
| If you would like to add resources or resource variables to the Material resources section, please use the box below. | | | | | | | | | | |  |
|  |  |  |  |  |  |  |  |  |  |  |  |
|  |  |  |  |  |  |  |  |  |  |  |  |
|  |  |  |  |  |  |  |  |  |  |  |  |
| **Human resources for a pandemic resource model** |  |  |  |  |  |  |  |  |  |  |  |
|  | Not relevant at all | | | | | |  |  | Extremely relevant | |  |
| Public Health | 1 | 2 | 3 | 4 | 5 | 6 | 7 | 8 | 9 | I don't know |  |
| Testing facility staff |  |  |  |  |  |  |  |  |  |  |  |
| Testing facility capacity |  |  |  |  |  |  |  |  |  |  |  |
| Contact tracing staff |  |  |  |  |  |  |  |  |  |  |  |
| Contact tracing rate |  |  |  |  |  |  |  |  |  |  |  |
|  | Not relevant at all | | | | | |  |  | Extremely relevant | |  |
| General hospital | 1 | 2 | 3 | 4 | 5 | 6 | 7 | 8 | 9 | I don't know |  |
| Laboratory testing capacity |  |  |  |  |  |  |  |  |  |  |  |
| Ambulance arrival time |  |  |  |  |  |  |  |  |  |  |  |
| Increase ambulance demand |  |  |  |  |  |  |  |  |  |  |  |
|  | Not relevant at all | | | | | |  |  | Extremely relevant | |  |
| Non-ICU | 1 | 2 | 3 | 4 | 5 | 6 | 7 | 8 | 9 | I don't know |  |
| Physicians per non-ICU bed |  |  |  |  |  |  |  |  |  |  |  |
| Nurses per non-ICU bed |  |  |  |  |  |  |  |  |  |  |  |
| Non-ICU physician absenteeism |  |  |  |  |  |  |  |  |  |  |  |
| Non-ICU nurse absenteeism |  |  |  |  |  |  |  |  |  |  |  |
|  | Not relevant at all | | | | | |  |  | Extremely relevant | |  |
| ICU | 1 | 2 | 3 | 4 | 5 | 6 | 7 | 8 | 9 | I don't know |  |
| Physicians per ICU bed |  |  |  |  |  |  |  |  |  |  |  |
| Nurses per ICU bed |  |  |  |  |  |  |  |  |  |  |  |
| ICU physician absenteeism |  |  |  |  |  |  |  |  |  |  |  |
| ICU nurse absenteeism |  |  |  |  |  |  |  |  |  |  |  |
|  |  |  |  |  |  |  |  |  |  |  |  |
| If you have any questions or remarks about the resources or resource variables above, please use the box below. | | | | | | | | | | |  |
|  |  |  |  |  |  |  |  |  |  |  |  |
|  |  |  |  |  |  |  |  |  |  |  |  |
| If you would like to add resources or resource variables to the Human resources section, please use the box below. | | | | | | | | | | |  |
|  |  |  |  |  |  |  |  |  |  |  |  |
|  |  |  |  |  |  |  |  |  |  |  |  |
|  |  |  |  |  |  |  |  |  |  |  |  |
| **Therapeutics for a pandemic resource model** | Not relevant at all | | | | | |  |  | Extremely relevant | |  |
|  | 1 | 2 | 3 | 4 | 5 | 6 | 7 | 8 | 9 | I don't know |  |
| Vaccine dose |  |  |  |  |  |  |  |  |  |  |  |
| Vaccine administration speed |  |  |  |  |  |  |  |  |  |  |  |
| Vaccine manufacturing capacity |  |  |  |  |  |  |  |  |  |  |  |
| Vaccine efficacy |  |  |  |  |  |  |  |  |  |  |  |
| Prophylactic antivirals dose |  |  |  |  |  |  |  |  |  |  |  |
| Prophylactic antivirals efficacy |  |  |  |  |  |  |  |  |  |  |  |
| Therapeutic antivirals dose |  |  |  |  |  |  |  |  |  |  |  |
| Therapeutic antivirals efficacy |  |  |  |  |  |  |  |  |  |  |  |
| Prophylactic antibiotics dose |  |  |  |  |  |  |  |  |  |  |  |
| Prophylactic antibiotics efficacy |  |  |  |  |  |  |  |  |  |  |  |
| Therapeutic antibiotics dose |  |  |  |  |  |  |  |  |  |  |  |
| Therapeutic antibiotics efficacy |  |  |  |  |  |  |  |  |  |  |  |
|  |  |  |  |  |  |  |  |  |  |  |  |
| If you have any questions or remarks about the resources or resource variables above, please use the box below. | | | | | | | | | | |  |
|  |  |  |  |  |  |  |  |  |  |  |  |
|  |  |  |  |  |  |  |  |  |  |  |  |
| If you would like to add resources or resource variables to the Therapeutics resources section, please use the box below. | | | | | | | | | | |  |
|  |  |  |  |  |  |  |  |  |  |  |  |
|  |  |  |  |  |  |  |  |  |  |  |  |
|  |  |  |  |  |  |  |  |  |  |  |  |
|  |  |  |  |  |  |  |  |  |  |  |  |
| Are there any other resources that you feel have been omitted in this survey? | | | | | | | |  |  |  |  |
|  |  |  |  |  |  |  |  |  |  |  |  |
|  |  |  |  |  |  |  |  |  |  |  |  |
| Has your experience of the COVID-19 pandemic changed your views on the role of resources in a pandemic? | | | | | | | | | | |  |
|  |  |  |  |  |  |  |  |  |  |  |  |
|  |  |  |  |  |  |  |  |  |  |  |  |
| Do you have any other comments with regard to this survey? | | |  |  |  |  |  |  |  |  |  |
|  |  |  |  |  |  |  |  |  |  |  |  |
|  |  |  |  |  |  |  |  |  |  |  |  |
|  |  |  |  |  |  |  |  |  |  |  |  |
| Thank you very much for participating in this survey, your contribution is greatly appreciated | | | | | | | | | | |  |

Supplementary material 3: Full outcomes of the Delphi consensus procedure with public health experts

|  | Delphi panel with public health experts | | | |
| --- | --- | --- | --- | --- |
|  | Pandemic planning | | Pandemic modelling | |
| Material resources | Questionnaire | Consensus | Questionnaire | Consensus |
| Total public health testing capacity | Accepted |  | Accepted |  |
| Testing reagents | Accepted |  | Discussion | Rejected |
| Test sensitivity | Discussion | Rejected | Accepted |  |
| Test specificity | Accepted |  | Accepted |  |
| Time to test result | Accepted |  | Accepted |  |
| Hospital PPE stock | Accepted |  | Accepted |  |
| PPE usage per bed per day | Accepted |  | Discussion | Accepted |
| PPE kit stockpile | Accepted |  | Discussion | Accepted |
| Conversion rate non-ICU to ICU | Accepted |  | Discussion | Accepted |
| Oxygen demand per bed | Accepted |  | Accepted |  |
| Rate of mechanical ventilation use | Accepted |  | Accepted |  |
| Duration of mechanical ventilation use | Accepted |  | Accepted |  |
| X-ray/radiography increased demand | Discussion | Rejected | Rejected |  |
| Total non-ICU beds available | Accepted |  | Accepted |  |
| Non-ICU admission rate | Discussion | Accepted | Accepted |  |
| Non-ICU length of stay | Discussion | Accepted | Accepted |  |
| Total ICU beds available | Accepted |  | Accepted |  |
| ICU admission rate | Accepted |  | Accepted |  |
| ICU length of stay | Accepted |  | Accepted |  |
| ICU length of stay with mechanical ventilation | Accepted |  | Accepted |  |
| Home care PPE stock | Rejected |  | Rejected |  |
| Home care PPE usage | Rejected |  | Rejected |  |
| Home care rehabilitation oxygen | Discussion* | Rejected | Rejected |  |
|  |  |  |  |  |
| Human resources | Questionnaire | Consensus | Questionnaire | Consensus |
| Testing facility staff | Accepted |  | Accepted |  |
| Testing facility capacity | Accepted |  | Accepted |  |
| Contact tracing staff | Accepted |  | Accepted |  |
| Contact tracing rate | Accepted |  | Accepted |  |
| Laboratory testing capacity | Accepted |  | Accepted |  |
| Ambulance arrival time | Rejected |  | Rejected |  |
| Increased ambulance demand | Accepted |  | Accepted |  |
| Physicians per non-ICU bed | Discussion | Rejected | Accepted |  |
| Nurses per non-ICU bed | Accepted |  | Accepted |  |
| Non-ICU physician absenteeism | Rejected |  | Rejected |  |
| Non-ICU nurse absenteeism | Accepted |  | Rejected |  |
| Physicians per ICU bed | Accepted |  | Accepted |  |
| Nurses per ICU bed | Accepted |  | Accepted |  |
| ICU physician absenteeism | Accepted |  | Accepted |  |
| ICU nurse absenteeism | Accepted |  | Accepted |  |
| Home care rehabilitation nursing staff | Rejected* |  | Rejected |  |
| Home care rehabilitation physiotherapy | Rejected |  | Discussion | Rejected |
| Home care nursing staff | Accepted* |  | Discussion | Accepted |
|  |  |  |  |  |
| Therapeutics | Questionnaire | Consensus | Questionnaire | Consensus |
| Vaccine dose | Accepted |  | Accepted |  |
| Vaccination administration speed | Accepted |  | Accepted |  |
| Vaccine manufacturing capacity | Accepted |  | Accepted |  |
| Vaccine efficacy | Accepted |  | Accepted |  |
| Prophylactic antivirals dose | Rejected |  | Discussion | Rejected |
| Prophylactic antivirals efficacy | Accepted |  | Accepted |  |
| Therapeutic antivirals dose | Accepted* |  | Discussion | Rejected |
| Therapeutic antivirals efficacy | Accepted |  | Accepted |  |
| Prophylactic antibiotics dose | Rejected |  | Rejected |  |
| Prophylactic antibiotics efficacy | Rejected* |  | Accepted |  |
| Therapeutic antibiotics dose | Rejected* |  | Rejected |  |
| Therapeutic antibiotics efficacy | Accepted |  | Accepted |  |
| *Outcome of internal discussion |  |  |  |  |

Supplementary material 4: Full outcomes of the Delphi consensus procedure with clinicians

|  | Delphi panel with clinicians | | | |
| --- | --- | --- | --- | --- |
|  | Pandemic planning | | Pandemic modelling | |
| Material resources | Questionnaire | Consensus | Questionnaire | Consensus |
| Total public health testing capacity | Accepted |  | Accepted |  |
| Testing reagents | Accepted |  | Accepted |  |
| Test sensitivity | Accepted |  | Accepted |  |
| Test specificity | Accepted |  | Accepted |  |
| Time to test result | Accepted |  | Accepted |  |
| Hospital PPE stock | Accepted |  | Accepted |  |
| PPE usage per bed per day | Accepted |  | Accepted |  |
| PPE kit stockpile | Accepted |  | Accepted |  |
| Conversion rate non-ICU to ICU | Accepted |  | Rejected |  |
| Oxygen demand per bed | Accepted |  | Rejected |  |
| Rate of mechanical ventilation use | Accepted |  | Accepted |  |
| Duration of mechanical ventilation use | Accepted |  | Accepted |  |
| X-ray/radiography increased demand | Rejected |  | Rejected |  |
| Total non-ICU beds available | Accepted |  | Accepted |  |
| Non-ICU admission rate | Accepted |  | Accepted |  |
| Non-ICU length of stay | Accepted |  | Accepted |  |
| Total ICU beds available | Accepted |  | Accepted |  |
| ICU admission rate | Accepted |  | Accepted |  |
| ICU length of stay | Accepted |  | Accepted |  |
| ICU length of stay with mechanical ventilation | Accepted |  | Accepted |  |
| Home care PPE stock | Accepted |  | Accepted |  |
| Home care PPE usage | Accepted |  | Accepted |  |
| Home care rehabilitation oxygen | Rejected |  | Rejected |  |
|  |  |  |  |  |
| Human resources | Questionnaire | Consensus | Questionnaire | Consensus |
| Testing facility staff | Accepted |  | Accepted |  |
| Testing facility capacity | Accepted |  | Accepted |  |
| Contact tracing staff | Accepted |  | Accepted |  |
| Contact tracing rate | Accepted |  | Accepted |  |
| Laboratory testing capacity | Accepted |  | Accepted |  |
| Ambulance arrival time | Discussion | Rejected | Discussion | Rejected |
| Increased ambulance demand | Discussion | Accepted | Accepted |  |
| Physicians per non-ICU bed | Accepted |  | Accepted* |  |
| Nurses per non-ICU bed | Accepted |  | Accepted |  |
| Non-ICU physician absenteeism | Rejected |  | Discussion* | Accepted |
| Non-ICU nurse absenteeism | Rejected |  | Accepted* |  |
| Physicians per ICU bed | Accepted |  | Accepted |  |
| Nurses per ICU bed | Accepted |  | Accepted |  |
| ICU physician absenteeism | Accepted* |  | Accepted |  |
| ICU nurse absenteeism | Accepted* |  | Accepted |  |
| Home care rehabilitation nursing staff | Accepted |  | Accepted |  |
| Home care rehabilitation physiotherapy | Rejected |  | Rejected |  |
| Home care nursing staff | Accepted |  | Accepted |  |
|  |  |  |  |  |
| Therapeutics | Questionnaire | Consensus | Questionnaire | Consensus |
| Vaccine dose | Discussion | Accepted | Accepted |  |
| Vaccination administration speed | Accepted |  | Accepted |  |
| Vaccine manufacturing capacity | Accepted |  | Accepted |  |
| Vaccine efficacy | Accepted |  | Accepted |  |
| Prophylactic antivirals dose | Rejected |  | Rejected |  |
| Prophylactic antivirals efficacy | Accepted* |  | Accepted |  |
| Therapeutic antivirals dose | Discussion | Accepted | Accepted* |  |
| Therapeutic antivirals efficacy | Accepted |  | Accepted |  |
| Prophylactic antibiotics dose | Rejected |  | Rejected |  |
| Prophylactic antibiotics efficacy | Rejected |  | Rejected |  |
| Therapeutic antibiotics dose | Rejected |  | Rejected |  |
| Therapeutic antibiotics efficacy | Discussion | Rejected | Rejected |  |
| *Outcome of internal discussion |  |  |  |  |
