# Supplementary material for: Identifying essential resource parameters for pandemic preparedness and response: an international Delphi study within the EU PANDEM-2 project
Source: BMJ Open. 2024 Dec 15;14(12):e079609. doi: 10.1136/bmjopen-2023-079609 (PMC11647290; doi:10.1136/bmjopen-2023-079609)
Supplement: Supplementary file 2 [file bmjopen-2023-079609.reviewer_comments.pdf]

PEER REVIEW HISTORY

BMJ Open publishes all reviews undertaken for accepted manuscripts. Reviewers are asked to complete a checklist review form and are provided with free text boxes to elaborate on their assessment. These free text comments are reproduced below.

ARTICLE DETAILS

Title (Provisional)

Identifying essential resources for pandemic preparedness and response: an international Delphi study within the EU-PANDEM-2 project

Authors

Beishuizen, Berend Hitjo Herman; Stein, Mart; Buis, Joeri; Tostmann, Alma; Green, Caroline; Duggan, James; Connolly, Máire; Rovers, Chantal; Timen, Aura

---

VERSION 1 - REVIEW

**Reviewer**	**1**
**Name**	**Piaggio, Davide**
**Affiliation**	**University of Warwick**
**Date**	**13-Oct-2023**
**COI**	**None**

This interesting piece of works builds on the results of a systematic literature review which will be published separately. It would have been great to have the review published first but I understand it may be challenging. The topic is very current and of interest. The authors use the Delphi study technique to find consensus on priority resources for Pandemic preparedness.

This work is well aligned with and complementary to a review my team and I have recently published on pandemic preparedness and governance from the policymaking point of view (https://www.mdpi.com/2227-9032/11/18/2572). Thank you for giving me the opportunity to learn more about this.

Overall the paper reads well and is well organised. Few suggestions and comments:

1) Validity is an important dimension when developing questionnaires. Did you look into this? If so, it would be great if you could mention it in the methods and results.

2) Similarly with reliability. This is important if you cluster items within bigger domains. It may be useful to look into that for your work.

3) You could also easily apply MCDA techniques to have a final ranking of the items included in this consesus list. As well as you could look into what items are correlated or not.

For points 1, 2, 3 and how to work them out, you could refer to our publication regarding another delphi survey:
https://globalizationandhealth.biomedcentral.com/articles/10.1186/s12992-021-00718-z

4) define abbreviations when first used (e.g., NGO, ICU...)

5) use the correct notation: decimals separeted by "." not "," e.g., 6.5 not 6,5

6)table 2 is cited after in the text compared to table 3, you should swap them?

7) Table 3, please spell patient as "patient", without the special e

Thank you.

VERSION 1 - AUTHOR RESPONSE

Reviewer 1

This interesting piece of works builds on the results of a systematic literature review which will be published separately. It would have been great to have the review published first but I understand it may be challenging. The topic is very current and of interest. The authors use the Delphi study technique to find consensus on priority resources for Pandemic preparedness.
This work is well aligned with and complementary to a review my team and I have recently published on pandemic preparedness and governance from the policymaking point of view (https://www.mdpi.com/2227-9032/11/18/2572). Thank you for giving me the opportunity to learn more about this.

Overall the paper reads well and is well organised. Few suggestions and comments:

We thank the reviewer for their valuable comments and suggestions, and compliments on readability and structure.

R1-1 Validity is an important dimension when developing questionnaires. Did you look into this? If so, it would be great if you could mention it in the methods and results.

Amended. Yes, we considered construct validity, content validity, and face validity when developing and piloting the questionnaire. Regarding construct validity, our questionnaire did not include complex constructs or items but did encompass commonly used resources or resource parameters. For content and face validity, we piloted our questionnaire internally with public health experts and clinicians, and externally in two rounds with a hospital capacity manager. After piloting, we added mouse-over explanations for some less obvious resource parameters, to the questionnaire to ensure the meaning was understood by all participants. We did not perform other tests to assess validity. A section titled **Validity** has been added to the methods on page 7 to explain this:

> *"We considered construct validity, content validity and face validity when developing the questionnaire. Construct validity was not considered to be an issue in this questionnaire as the items included do not represent or reflect concepts, but instead are real resources. Mouse-over explanations were added to the questionnaire to support construct validity. We piloted the questionnaire for content and face validity, first internally with public health experts and clinicians of diverse background, and secondly externally in two rounds with a hospital capacity manager."*

R1-2 Similarly with reliability. This is important if you cluster items within bigger domains. It may be useful to look into that for your work.

We agree that reliability is important when using questionnaires in research. We have now calculated the Cronbach's alpha (using IBM SPSS) to check retrospectively and found that Cronbach's alpha was 0.899 indicating great to excellent internal consistency of the questionnaire as a whole, and per domain 0.913 for material, 0.899 for human and 0.721 for pharmaceutical resources.

R1-3 You could also easily apply MCDA techniques to have a final ranking of the items included in this consensus list. As well as you could look into what items are correlated or not.
For points 1, 2, 3 and how to work them out, you could refer to our publication regarding another delphi survey: https://globalizationandhealth.biomedcentral.com/articles/10.1186/s12992-021-00718-z

We thank the reviewer for this suggestion. MCDA techniques and ranking of items can be a useful tool in Delphi studies, but it their relevance depends on the study design and aim. In the linked study (https://globalizationandhealth.biomedcentral.com/articles/10.1186/s12992-021-00718-z) a hierarchy framework was used, which makes the ranking of items evaluated very relevant. However, in our study we did not use a hierarchy framework and did not intend to draw conclusions that certain resources are more relevant than others, and also our study was not conducted with low-resource/scarcity settings in mind. The outcomes of our study can be used to inform pandemic preparedness planning, or resource modelling for preparedness. Both allow for inclusion of a large number of resources. Instead of ranking (for example using the Relative Index), we employed a cut-off value for median score and level of agreement to classify the resources, as also used in Belfroid et al. (Towards defining core principles of public health emergency preparedness: scoping review and Delphi consultation among European Union country experts. BMC Public Health. 2020;20(1):1482, https://link.springer.com/article/10.1186/s12889-020-09307-y). This is explained in section **2.5 Data analysis** on page 7.

We looked into correlation within domains ('material resources', 'human resources', and 'pharmaceutical resources') of included items using Pearson correlation calculated with SPSS (Outcomes for material resources given in the table below). We found that certain resources correlate strongly with each other as expected based on their practical relations (Total non-ICU beds available correlating with non-ICU bed admission rate, non-ICU bed length of stay and ICU bed length of stay). Some resources correlated with very few others (or not at all, for Total ICU beds available). However, we believe that a study such as ours can be assessed only through descriptive statistics and empirical judgment, because the data are not relatable to some form of random sampling and are therefore not amenable to statistical inference, meaning that such things as p-values and confidence intervals have no real meaning and that, for example, even correlations between items can only be judged subjectively – which is not a problem at all since this is just the type of judgment that, in the final analysis, is needed in decision making.

Item	Correlation ($-1>R>-0.5$ & $0.5<R<1$, $p < 0.05$)					
public health testing	non-icu admission rate					
test specificity	duration mech vent use					
time to test result	non-icu los	icu admission rate	icu los	icu los mech vent		

hospital ppe stock	total non-icu available	non-icu admission rate	non-icu los	icu los				
ppe usage per bed per day	ppe kit stockpile							
ppe kit stockpile	ppe usage per bed per day	rate mech vent use						
rate mech vent use	ppe kit stockpile	rate mech vent use	icu los	icu los mech vent				
duration mech vent use	test specificity	rate mech vent use	non-icu los	icu los mech vent				
total non-icu available	hospital ppe stock	non-icu admission rate	non-icu los	icu los				
non-icu admission rate	public health testing	hospital ppe stock	total non-icu available	non-icu los	icu los			
non-icu los	time to test result	hospital ppe stock	duration mech vent use	total non-icu available	non-icu admission rate	icu los	icu los mech vent	
total icu available								
icu admission rate	time to test result	icu los	icu los mech vent					
icu los	time to test result	hospital ppe stock	rate mech vent use	total non-icu available	non-icu admission rate	non-icu los	icu admission rate	icu los mech vent
icu los mech vent	time to test result	rate mech vent use	duration mech vent use	non-icu los	icu admission rate	icu los		

R1-4 define abbreviations when first used (e.g., NGO, ICU...)

Amended.

R1-5 use the correct notation: decimals separeted by "." not "," e.g., 6.5 not 6,5

Amended.

R1-6 table 2 is cited after in the text compared to table 3, you should swap them?

Amended.

R1-7 Table 3, please spell patient as "patient", without the special e

Amended.

Reviewer 2

We thank the reviewer for their critical and insightful comments on our manuscript.

R2-1 The paper uses a fairly standard Delphi consisting of a systematic literature review, a questionnaire and two international expert panels (conducted in January, February and May 2022). the manuscript reads as if the questionnaire was piloted twice but only with a single hospital resource management specialist? Would it not have been preferable to ensure clarity of the questions to pilot with several people, both clinicians and PHE experts? The number of resources suggested by respondents (table 3) suggests the piloting was inadequate. The reference 16 to Delphi methods is 23 years old and pre-dates the use of online participation (Rand used face to face panels with relatively small numbers) - Fitch K, Bernstein SJ, Aguilar MD, Burnand B, LaCalle JR. The RAND/UCLA appropriateness method user's manual. Rand Corp Santa Monica CA; 2001. More recently, Equator publish reporting guidelines for publications using Delphi methods - https://www.equator-network.org/wp-content/uploads/2009/02/Protocol-E-Delphi-studies-reporting-guideline.pdf

Amended. Yes, we considered construct validity, content validity, and face validity when developing and piloting the questionnaire. Regarding construct validity, our questionnaire did not include complex constructs or items but well known resources or resource parameters. For some less obvious resource parameters, we added mouse-over explanations to the questionnaire to make sure the meaning was understood by all participants. For content and face validity, we piloted our questionnaire internally with public health experts and clinicians, and externally in two rounds with a hospital capacity manager. We did not perform other tests to assess validity. A section titled **Validity** has been added to the methods on page 7 to explain this. The number of resources suggested may reflect suboptimal content validity, however we do not consider this an issue

Yes, we chose to include the reference to the RAND/UCLA appropriateness method as they originally developed the Delphi method, but the study design was based mostly on a recently conducted Delphi study by colleagues (Belfroid E, Rosskamp D, Fraser G, Swaan C, Timen A. Towards defining core principles of public health emergency preparedness: scoping review and Delphi consultation among European Union country experts. BMC Public Health. 2020;20(1):1482, https://link.springer.com/article/10.1186/s12889-020-09307-y). We also draw on Hsu and Sandford's very practical paper Hsu | The Delphi Technique: Making Sense of Consensus | Practical Assessment, Research, and Evaluation (umass.edu). We have used the CREDES reporting guidelines for this study but realise that it was omitted from the supplementary material, this has been **amended**.

R2-2 Why did initial recruitment targeted at public health and clinical care professionals yield only public health experts? This failure led to 2 separate consensus strategies and then a review of the commonality between them. Two online consensus rounds were held to discuss those items for which no consensus was reached in the questionnaire. Does this mean 2 rounds, one for clinicians and one for PHE, or 2 rounds in series with all respondents in the meeting? One of the reasons for convening a consensus meeting after the questionnaire stages is so that respondents who have graded statements differently can hear others' points of view. If the 2 rounds were one for clinicians and one for PHE, then this benefit is lost and there is a risk of 'groupthink', all attendees from the same discipline reinforcing their preconceived Likert scores in an 'echo chamber'. In Section 2.4 the explanation "These personal reports showed the scores given to each resource by the entire panel, the score given by the respondent in question, outcomes of internal discussion and new suggestions. Due to the sequential nature of both consensus procedures, new suggestions were only discussed within the respective panel." seems very ambiguous to me as a reader. The methods must be more explicit re whether the authors were essentially running 2 completely separate Delphi exercises or they were somehow combined at some point? "This procedure was held for the two expert panels mentioned above: PHEs (27th January and 14th February 2022; two sessions needed) and clinicians (30th May 2022)" reads to me like 2 completely separate Delphi exercises. The reader has to wait until reaching the Discussion to fully appreciate "Our Delphi study was conducted with two separate international panels. Our initial intentions were to conduct one Delphi with a mixed panel of both PHEs and clinicians, however this was adapted as our initial recruitment yielded only PHEs."

Clarification. We recruited participants using the purposive snowball sampling strategy, targeting specific individuals for recruitment and asking them to forward the invitation to other experts in their network, explicitly requesting participants working in public health or a clinical setting with experience in pandemic preparedness and response. For international participants, we asked partners in the PANDEM-2 project, of which this study was a part, to recruit in their network. We started recruitment early in the fall of 2021, just before the Omicron variant resulted in a large new wave of COVID-19 infections. We suspect that this significantly impacted our recruitment, with

clinicians not being able to participate. Because we had recruited a sufficient number of public health experts to go ahead with a consensus round, the decision was made not to delay the study but start with public health experts, and then attempt to recruit a second panel of clinicians in the spring of 2022, when the pressure of COVID-19 cases was reduced. We have added clarification for this in the discussion of **strengths and limitations** at lines 306-313.

> *"Our Delphi study was conducted with two separate international panels. Our initial intentions were to conduct one Delphi with a mixed panel of both PHEs and clinicians, however this was adapted as our initial recruitment yielded only PHEs. A probable cause for the initial failure to recruit clinicians lies in the timing: recruitment was started in October 2021, just before the Omicron variant emerged and resulted in a large COVID-19 wave in Europe. The resulting increased hospitalisation rate and clinical load may have prevented clinicians from participating in our study. Sufficient PHEs were recruited to start a consensus round and in the of 2022, when the pressure of COVID-19 cases was reduced, a panel of clinicians was recruited for the second consensus round."*

Regarding the risk of 'groupthink' or an echo chamber effect occurring because we conducted separate panels and were not able to mix public health experts and clinicians, we believe we sufficiently mitigated during the online consensus meeting this through moderation, where moderators explicitly asked participants to give their individual opinions before discussing, and use of a blind voting system.

The explanation on personal reports in section 2.5 Consensus procedures has been rephrased to be less ambiguous at lines 169-172.

> *"Prior to the consensus round, each participant received a personal feedback report detailing the analysis of the scores, their personal scores for each resource and an overview of the group scores. Within the separate panels, additional resources suggested in open text fields were added to the report."*

It is correct that due to these practicalities we conducted two separate Delphi procedures, and then compared the findings afterwards. To make sure this is clear before reading the results, we have emphasized this in **2.1 Study design** at lines 107-109.

> *"This study utilizes a Delphi consensus procedure [17, 18] consisting of a systematic literature review, a questionnaire and two separate international expert panels (the first conducted in January and, February 2022, and the second in May 2022)."*

And also at **2.6 Consensus procedures** at lines 179-182.

> *"This procedure was held for the two expert panels mentioned above: first a consensus procedure with PHEs (27th January and 14th February 2022; two sessions needed) and a separate consensus procedure with clinicians (30th May 2022)."*

R2-3 "The panel of PHEs included participants from Austria, Finland, Germany, Ireland, Italy, the Netherlands, Portugal, Romania and Sweden; the panel of clinicians included participants from Finland, Ireland, the Netherlands and Portugal." Surely this makes comparisons between the consensus of clinicians and PHE experts very difficult. PHE views reflect 9 countries including 2 with large populations. Clinicians came from only 4 smaller countries. Moreover, the experience respondents will have drawn on will have been very different between the 5 countries omitted and the 4 included given the variation in political and social decisions by different nations. Sweden is an

obvious outlier in this respect. Perhaps this explains some of the “unexpected findings”. Eg “the conversion rate from non-ICU to ICU beds was expected to be important for pandemic preparedness planning. However, the panel with clinicians rejected this resource for pandemic preparedness planning. Ventilator demand was also high during COVID-19, which in turn caused a high demand for oxygen. Oxygen shortages have been reported, especially in low- and middle-income countries. Notably though, clinicians rejected the resources 'conversion rate non-ICU to ICU' and 'oxygen demand per bed' for pandemic preparedness planning, while these were accepted by the PHEs”.

Clarification. Yes, we agree that not having the exact same backgrounds in both panels is a limitation with regard to comparison of outcomes. However, differences in views may also occur within a country. We believe that some of the unexpected findings are more due to differences in professional background and perspective between public health experts and clinicians rather than different countries of origin. There was a high degree of consensus between the two expert panels, and the set of resources that both panels agree on is valuable as it is supported by experts from varied European backgrounds and thus more applicable to other European countries.

R2-4 The respondents had a median of 3 years’ experience. This seems skewed towards a very limited experience. Why?

Clarification. As explained above (see R2-3), we recruited respondents through partners in a European research project and thus did not have full control over responses. To clarify, the years’ experience in the panel with public health experts was indeed somewhat skewed, resulting in a low median. In fact nine respondents had <5 years’ experience, two had between 5 and 10 years’ experience, and six had >=10 years’ experience, providing sufficient depth to that panel. In the panel with clinicians, the median was 6.5 years’ experience and the average was 8.2 years, providing sufficient depth. Unfortunately, some of the more experienced clinicians were only able to take part in the questionnaire phase and not the consensus meeting resulting in a lower median/average years’ experience. We have added the averages to the participant characteristics in table 1 for clarification.

R2-5 Did the authors consider seeking the views of other stakeholders, eg politicians or the public. Personally, I don’t think this is critical to this publication but a factor during the pandemic was the dissonance sometimes between technical healthcare advice and political, financial and social judgements. Was there any PPIE in study design or questionnaire content?

Public and patients, or politicians were not included in the design of this study, this is also stated in section **2.8 Patient and public involvement (page 9)**. We did collect input on the study design from other colleagues involved in policy advice and pandemic preparedness policy. However, it can be said that all involved stakeholders approached the subject from a public health or healthcare perspective during the then ongoing pandemic. We agree with the reviewers’ comment that during a pandemic not only the technical healthcare aspects but social and economic aspects should also be considered when making policy decisions. With this study we seek to specifically contribute to pandemic preparedness and technical healthcare advice.

R2-6 Were the respondents given indicative costs of each item they were asked to score and expiry times after which drugs, PPE, consumables etc would need to be procured afresh? It is easy for clinicians and PHE experts, and the public, to expect a comprehensive pandemic preparedness but the items need to be weighed against the likelihood of use and the % of finite healthcare expenditure diverted away from other uses.

Clarification. No, we did not provide indicative costs of the items included in the study. We agree with the reviewers' comment that cost and shelf-life of are important when deciding which resources to procure and stockpile, however we consider this economic analysis beyond the scope of our study. The aim of our study is to determine which resources are considered most relevant for pandemic preparedness plans or pandemic resource modelling by the panels of public health experts and clinicians. The findings of our study provide input for the question which healthcare resources are needed, and resource modelling contributes to determining the quantities of healthcare resources that are required to maintain continuity of care in outbreak or pandemic scenarios. A subsequent step for stockpile and procurement planning will be to add resource costs and shelf-life to the equation and determine what is then feasible and possible within the constraints of pandemic emergency funds or hospital budgets. This involves policy decisions and is beyond the scope of this work. We mention in the introduction that financial assets are not part of the scope of this study, we have expanded this to include resource cost to clarify at **lines 79-80:**

> *"Financial assets are a vital resource as well, and the cost and shelf-life of resources are important aspects for pandemic resource planning, but neither are part of the scope of this study."*

R2-7 Table 2 are not resources as I understand them. These are metrics of healthcare provision. Eg the resources required in a pandemic would be a contact tracing system, antibiotics, nurses and the metrics used to asses a system's preparedness might be number of Contact tracing staff per million population, number of antibiotic doses stockpiled per million population, Nurses per ICU bed etc. It wasn't until I reached p8 of the MS and Table 2 that I realised my expectation that this paper would shed light on "Identifying essential resources for pandemic preparedness" was misplaced. The authors were not seeking consensus on what would be the expected number of Contact tracing staff per million population, number of antibiotic doses stockpiled per million population, Nurses per ICU bed etc to deal with a future pandemic but seeking consensus on what would be the metrics. In this sense, the title, abstract and intro need to be clearer.

Clarified. The reviewer is correct in observing that we look at the metrics (or parameters) of resources in this study. We conducted a separate literature review (also currently under review) providing an overview of public health and healthcare resource data published in scientific literature in relation to pandemic influenza and the COVID-19 pandemic. This resulted in a list of (and specific data for) many different resource types and associated parameters or metrics which give value to the demand or use of a resource in practice. For example, ICU beds are a resource, and the parameters 'ICU admission rate', 'ICU length of stay' and 'ICU length of stay with mechanical ventilation' determine how many ICU beds are needed. These resource parameters are the data that pandemic planners or resource modellers require as input. Thus, the aim of our study was to determine which of the resources and associated parameters are most relevant for pandemic preparedness and resource modelling for pandemic preparedness, and not to determine the specific amount of resource that might be needed, because that is very context dependent and not generalizable. We have clarified this in the title and abstract by specifying resource parameters and in the **introduction** at lines 73-78 & 86-89, and note that throughout the remainder of the manuscript we refer to resources and their parameters collectively as 'resources':

> *"Healthcare resources can be loosely defined as the people and items required to keep all parts of the healthcare system (primary care, clinical care, home care and also public health) running. For pandemic preparedness or resource modelling for pandemic preparedness not only the resource (e.g. physicians) but also the associated parameters (e.g. number of beds a physician attends, rate of physician absenteeism) are needed to determine the rate at which*

a resource is used and thus how many are needed to control infectious diseases and maintain care delivery."

"Expanding models to include more resources and resource parameters (from here on collectively referred to as 'resources'), and both clinical care and public health resources, allows simulation of more realistic scenarios for preparedness exercises."

VERSION 2 - REVIEW

**Reviewer**	**1**
**Name**	**Piaggio, Davide**
**Affiliation**	**University of Warwick**
**Date**	**03-Oct-2024**
**COI**	

Thank you for solving all my doubts and comments. I really appreciated your hard work and thorough explanations!

VERSION 2 - AUTHOR RESPONSE

Reviewer 2 comment 3: The panel of PHEs included participants from Austria, Finland, Germany, Ireland, Italy, the Netherlands, Portugal, Romania and Sweden; the panel of clinicians included participants from Finland, Ireland, the Netherlands and Portugal." Surely this makes comparisons between the consensus of clinicians and PHE experts very difficult.

Response:
not having the exact same backgrounds in both panels is a limitation with regard to comparison of outcomes. However, differences in views may also occur within a country. We believe that some of the unexpected findings are more due to differences in professional background and perspective between public health experts and clinicians rather than different countries of origin. There was a high degree of consensus between the two expert panels, and the set of resources that both panels agree on is valuable as it is supported by experts from varied European backgrounds and thus more applicable to other European countries.

Amended. Lines 326-333:

"Both panels did not have the exact same composition with regard to country of employment, and the panel of clinicians featured higher representation from the Netherlands and Finland. This may be a limitation with regard to comparison of outcomes and result in a possible bias towards outcomes relevant for those countries. No country was overrepresented in the panel of PHEs, thus reducing the risk of bias in that regard. However, differences in views may also occur within a country, and we believe that some of the unexpected findings are more due to differences in professional background and perspective between public health experts and clinicians rather than different countries of origin."
